# Effect of Geometry and Size on Additively Manufactured Short-Fiber Carbon-Nylon Composite Under Tensile Loading

**DOI:** 10.3390/polym17030401

**Published:** 2025-02-03

**Authors:** András Kámán, Armand Meszlényi, Miklós Jakab, András Kovács, Attila Egedy

**Affiliations:** 1 Department of Process Engineering, Faculty of Engineering, University of Pannonia, 8200 Veszprém, Hungary; meszlenyi.armand@mk.uni-pannon.hu (A.M.); egedya@fmt.uni-pannon.hu (A.E.); 2 Department of Material Sciences, Faculty of Engineering, University of Pannonia, 8200 Veszprém, Hungary; jakab.miklos@mk.uni-pannon.hu (M.J.); kovacs.andras@mk.uni-pannon.hu (A.K.)

**Keywords:** 3D printing, nylon composites, specimen geometry, tensile strength, CT scan

## Abstract

As the articles relating to the study of 3D printing processes are picking up pace, the question of comparability and repeatability based on the geometry and size of the specimens arises, based on the fact that the widely used extrusion 3D printing processes inherently have a structure that is made up of extruded lines of various shapes and sizes. This study aimed to determine the impact the specimen geometry and size have on the final tensile strength. One of the most widely used engineering materials, chopped carbon-fiber-reinforced nylon was used for this study. The four main specimen groups examined were specimens containing only walls and specimens containing only infill printed with both a 0.4 mm and 0.8 mm nozzle (to determine that the size of the extrusion lines has any effect on the tensile strength with different specimen sizes) achieving a solid body with two different line structures. Contradictory to the initial expectations, the tests showed that the geometry and size of the specimens had not influenced the tensile strength of the specimens in any of the four specimen groups. However, the tests showed that the groups containing only walls were always stronger than their only-infill counterparts and the groups printed with a 0.4 mm nozzle were stronger than the groups printed with a 0.8 mm nozzle.

## 1. Introduction

Fiber-reinforced 3D printing is an emerging area with a wide range of possibilities not only in the field of engineering but also in medical or consumer fields (Tuli et al., 2024 [1]). There are several different 3D printing methods (FDM, SLA, SLS, Binder jet etc.); however, this paper focuses on FDM/FFM printing because it is the most widely used and customizable additive manufacturing method. Natural and bio inspired fibers and matrix compounds as well as synthetic ones can be incorporated. However, most of the applied matrix compounds are PLA or nylon due to their superior characteristics (Jamal et al., 2024 [2]). The applied fibers should increase some of the characteristics (mechanical, thermal, and elastic properties); however, we should keep in mind that for a detailed comparison, a proper optimisation of the printing parameters and the use of adequate printers and parameters is key to achieving an optimal product. Besides polymer 3D printing, other emerging fields are using similar manufacturing processes (such as extrusion-based FDM).

- Geopolymers (Fatheali et al., 2023 [3]).
- Cement including rubber particles (Zhu et al., 2023 [4]).
- Cement including fibers (Pi et al., 2024 [5], Asghari et al., 2024 [6]).
- Metal 3D printing (Clarke et al., 2023 [7]).
- Polymer-ceramic filaments (Podgorski et al., 2023 [8]).

There are two main reinforcement types in extrusion-based 3D printing, those being continuous- and short-chopped fibers. Both types of fibers enhance specific material properties; however, their behaviour can slightly differ. Also, continuous-fiber printing is still in an infantile state and can only be performed with specifically built DIY printers and expensive industrial or semi-industrial printers. In contrast, chopped-fiber-reinforced 3D printing can be carried out in any commercially available printer (besides the abrasion-resistant nozzle) and the prices for these materials usually stop at about twice the price of the pure matrix material; for these reasons, this study solely focuses on the chopped-fiber-reinforced material.

There have been an increasing number of studies relating to 3D printing such as Sun et al., 2023 [9], who used continuous-fiber-reinforced composites and calculated the optimal turning radius for nylon–carbon fiber systems. A 7 mm fiber was used and eSun PA6/1.75 mm nylon with a robot-based system. They created a near-perfect fiber-turning model and validated the model against measurement data. Zhang et al., 2024 examined the conservative lower bound for strengths of fused filament fabrication and selective laser sintering. eSun PLA filaments were used for the FFF, while PA12 was used for the SLS experiments. Based on the results, they created a new quadratic strength criterion. Wang et al., 2024 [10], proposed a novel topology optimisation for continuous-fiber 3D printing. With the optimised structure, better microstructure and stress distribution can be achieved while avoiding the possible defects (overlapping, sharp corners, insufficient infill). Long et al., 2022 [11], aimed to improve the mechanical properties of continuous-fiber composites by optimisation. They focused on printing efficiency and maintaining acceptable tensile strengths. PLA and flax yarn fibers were applied, and the printing speed and printing line width were used in a Response Surface Optimisation method; the printing efficiency resulted in a 40 percent increase. Ding et al., 2023 [12] examined the effect of printing design on the pseudo ductility in continuous-fiber-reinforced glass/carbon nylon composites. They found out that the layer thickness has no effect on the pseudo-ductility, while the printing platform temperature can decrease the defects generation. Liu et al., 2024 [13], created a finite element model to simulate the printing process focusing on the effects of pressure on the continuous-fiber-reinforced composites. They validated the model and they found out that the optimisation of the printing temperature and speed can eliminate the warping issues, and with the use of mathematical models, the optimisation process can be optimised. Peng et al., 2024 [14] examined the application of nacre-based topologies for the creation of carbon fiber composites. Optical and scanning microscopy was also included in the detection and evaluation of the microstructure. They concluded that the interlocking composites have superior performance, and multi-material FDM-based printing has vast opportunities in biomimetic applications.

Shi et al., 2021 [15] used kevlar reinforced nylon. Different fiber angles were tested, and the effect of printing on the mechanical properties were evaluated, as well as the autoclave treatment, which increased the stiffness by about 1 magnitude, mostly caused by the decrease of porosity. Ding et al., 2024 [16] focused on the effect of layer thickness and path wide as an effector of 3D printing of preimpregnated glass and cabron fibre composites, using Markforged filaments, preimpregnated whit nylon. They compared the glass and carbon reinforced composites together, and also created hybrid composites, which produced almost 200 percent increased flexular strength. Rimasauskas et al., 2022 [17] also investigated chopped and continuous carbon fiber reinforced composites. They point out that some of the applications, especially high demanding as aerospace sector the void fraction and fiber content are both critical, but challenging to measure. Layer height and line width are adjusted, while the void fraction are detected with computer tomography (CT). Between 18.5 and 27.5 percent of void fraction was found, which definitely influences the mechanical properties. Besides nylon and PLA there are still ABS based applications for composite structures.

Sun et al., 2023 [18] examined the different printing parameters for nylon composites with short carbon fibers. They also created the initial material from scratch, printing speed, infill structure and other parameters were also investigated. They also introduced a vacuum heat treatment process of different temperatures. CF content, raster angle, and print substrate temperature were investigated, and the effect of the heat treatment was also evaluated, using SEM images. However, the porosities were not changed due to the heat treatment. Furthermore, Calignano et al., 2020 [19] also investigated the mechanical properties of a carbon-fiber-reinforced nylon filament which included the tensile properties of samples with different infill ratios and build orientation. Moradi et al., 2024 [20] made a further contribution to the investigation of this material group with the optimisation of extruder temperature and printing speed regarding the tensile properties.

The main focus of this study was on the geometry and size of testing specimens as we have yet to find a paper that investigated this aspect. Almost all of the studies listed above have either lacked the precise documentation of printing parameters and methodology or given information about parameters that are irrelevant to the experiments as they are usually dependent on the specific material and specific printer used; one such example is a paper from Rybarczyk et al., 2024 [21], which investigated the mechanical properties of carbon-fiber-reinforced materials, including nylon. However, the printing parameters given are inconsequential with seemingly arbitrary values. Also, the number of papers that utilize CT imagining to determine cavities and porosity is low, so in order to contribute to this side of the 3D printing investigations, the porosity system has been studied with the intention of determining its effect on the tensile strength property.

## 2. Materials and Methods

The tensile test specimens were created using an extrusion 3D printing process, the used materials were chopped carbon-fiber-reinforced PA6/PA66 blend, which was chosen as it is one of the most widely used and affordable engineering filaments. This study focused on the two main load-carrying structures of 3D-printed objects. One of these is the only-infill group, in which the specimens only consist of alternating layers of parallel extruded lines, where the lines inside of a layer are perpendicular to the neighboring layer lines. This group represents the horizontal shells of a printed part, known also as the top and bottom layers. The other group is the only-wall specimens, in which all the layers are identical to one another; this group represents the vertical shell of printed parts, also known as wall, wall lines, or wall loops. The main difference between these groups is the orientation of the chopped fibers, as the fibers inside the extruded lines naturally orient themselves in the direction of the printhead movement (Kaman et al. 2024 [22] and Almeida et al. [23]); the only-infill group is made up of layers where half of them are oriented in the direction of the tensile testing and the other half perpendicular to it. In the only-wall group, all the layers and extruded lines in them are oriented in the direction of the tensile testing.

Also, all the 3D printing parameters of the specimens were the same, except for the nozzle diameter and structure. The two nozzle diameters used were the most widely used 0.4 mm and 0.8 mm nozzles in order to determine if the nozzle diameter has any effect on the overall impact of the specimen geometry as this parameter determines the size of the extruded lines a printed part is made up from. The two structures were the only-infill and only-wall structures, both achieving a 100% solid part which is homogeneous (usually a mix of both is used for printing resulting in a heterogeneous structure). The only-wall structure is made up of extruded lines, where all the layers are identical and all the extruded lines are parallel to the axis of the tensile testing, while the only-infill structure in a rectilinear pattern is made up of alternating layers, in which half of the layers contain extruded lines which are perpendicular to the axis of tensile testing and half is parallel.

**Figure 1:**
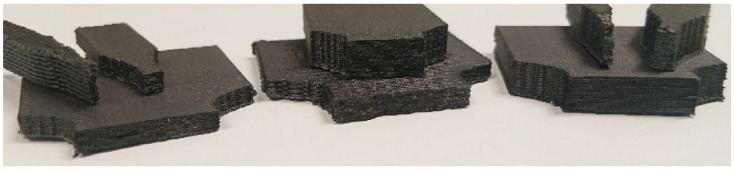
The visual difference in shape and size between individual specimens after tensile testing.

The width/height ratio of specimens ranged between a value of 0.22 and 8.5, while their cross-section area was between a value of 9.11 ${\mathrm{mm}}^{2}$ and 120.27 ${\mathrm{mm}}^{2}$. For better understanding of the difference between sizes and geometries of the specimen groups, they can be seen after testing in Figure 1.

### 2.1. 3D Printing Process

All the specimens were made on a Rat Rig V-Core 3 (Faro, Portugal) machine. It was chosen mainly for its modularity and quality mechanical components. Also, due to the fact that this printer is designed to be run on Klipper firmware (v.0.11.0) with RatOS (V 2.0.2), the possibility to tweak settings down to the most basic levels is available. For the mechanical parts, MGN12 linear rails with MGN12C carriages, a 6 mm thick aluminium bed and 30 mm × 30 mm aluminium extrusions make up the frame of the printer. For the material deposition part, the two main components are the Orbiter V1.5 extruder (Shenzhen, China) and Slice Engineering Mosquito hotend (Gainesville, FL, USA) with E3D V6 steel nozzle; the PTFE tubing used was a Capricorn PTFE tube. The Slicer used for the GCODE generation was PrusaSlicer (V 2.7.0) and all the non-adjusted parameters list can be seen in the Supplementary File. Before the specimen printing took place, the following calibrations were carried out to ensure the best results possible: extrusion multiplier, retraction length and speed, resonance compensation, pressure advance, skew correction. Some of the chief printing parameters were as follows

**Figure 2:**
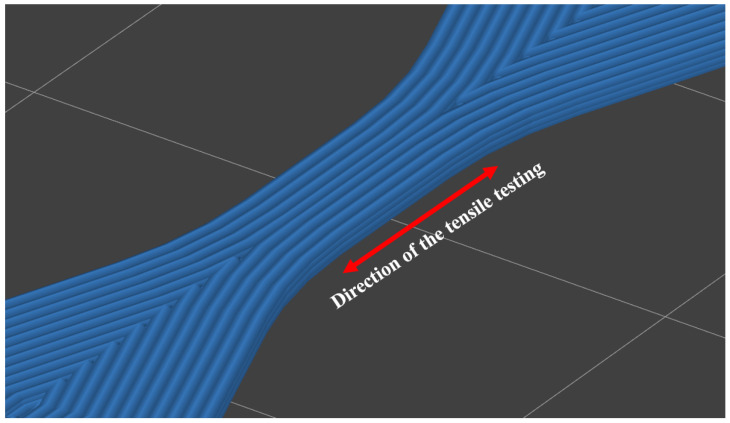
The relation between the infill orientation and tensile testing.

- A 50% layer height (realitve to the nozzle diameter).
- A 0° infill orientaion (relative to the direction of tensile testing, Figure 2).
- No top and bottom layer.
- Arachne perimeter generation.
- All printing speeds were set to 40 mm/s except the first layer for adhesion purposes, which was 15 mm/s.
- A 250 °C printing temperature.
- Part cooling disabled.

The materials used were eSun ePA-CF Natural (Shenzen, China), which is a nylon 6/nylon 66 blend with 20 wt% chopped carbon-fiber-reinforcement. Before printing was performed, the filaments were left in a food dehydrator at 70 °C for 24 h and they remained in it during the whole process of 3D printing in order to negate any moisture absorption of the nylon matrix (Hadi et al. [24] and Majko et al. [25]). The main properties of the filament can be observed in Table 1.

### 2.2. Tensile Testing

**Figure 3:**
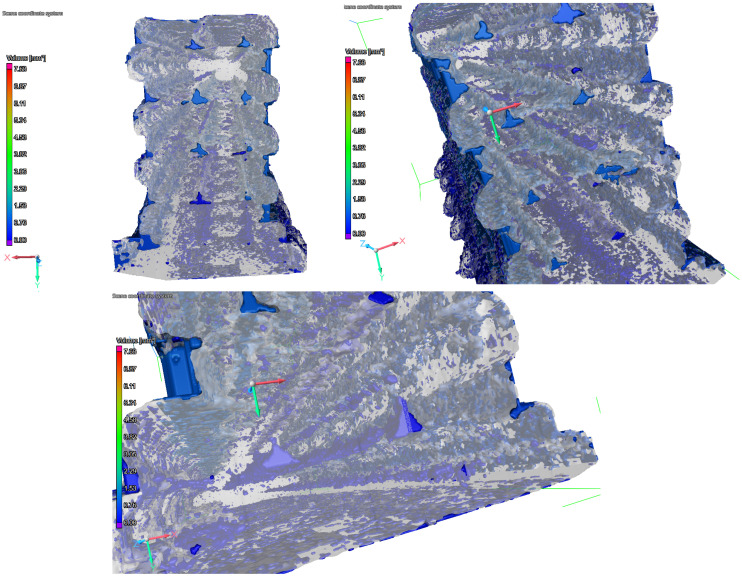
The porosity systems of an only-infill specimen captured in the CT imagining software.

ASTMD D638 type V specimens and their derivatives were compared with different width-to-height ratios as well as sizes in order to determine the effect these properties have on the tensile strength on the presumption that the nature of their extruded line-based structures can play a role during tensile stresses. All the tensile testing was carried out on an INSTRON 3385H universal testing machine (250 kN) (Norwood, MA, USA), with a 5 kN static load cell that made the measurements much more precise for forces that arise during the testing of the specimens. A group of specimens was also examined with Nikon XT H 225 ST X-ray computed tomography (Nikon Metrology Europe, Leuven, Belgium) before tensile testing, in order to determine the effect of porosity on the final mechanical property, the layout of different porosity systems have been observed in VGStudio Max software V.2023/1 (Volume Graphics, Heidelberg, Germany) (Figure 3).

## 3. Results

**Figure 4:**
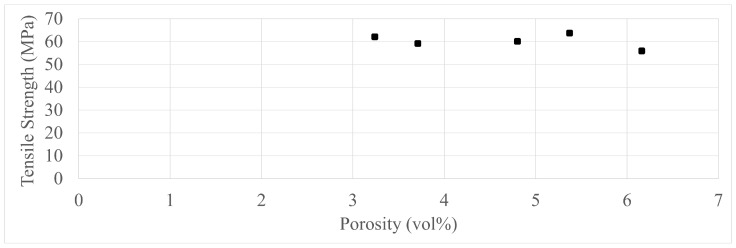
The tensile strengths of the pretest specimens compared by their porosity.

CF nylon specimens were tested in order to determine that the different porosities yield different strengths, such porosity systems can be seen from different angles in Figure 3. Five basic groups were tested with 0.4 nozzles and only infill (Table 2), with CT porosity analysis carried out before tensile testing. The Figure 4 shows that the porosity had no effect on tensile strength in this 3–6 vol% range, keeping in mind that material deposition was calibrated beforehand for this exact reason validating that the mentioned calibrations are a necessity.

The extrusion-based 3D-printed bodies are made up of lines of various shapes and sizes (chiefly determined by the nozzle diameter and layer height). Computed tomography results revealed that even with all the calibration carried out beforehand, the porosity of specimens could not be lower than 1.5 vol %, but in most cases, the porosity was between 3 and 6 vol%.

The specimens were compared by their cross-section area, width/height ratio, width/nozzle diameter, height/layer height. The meaning behind these geometric parameters are the following:

- Cross-section area indicates an overall bulk of a specimen.
- Width/height ratio indicates how the deposited material is distributed between layers and extruded lines inside of those layers.
- Width/nozzle diameter is simply the number of extruded lines inside a layer that is perpendicular to the tensile testing.
- Height/layer height gives the information of how many layers the specimens are made up of.

**Figure 5:**
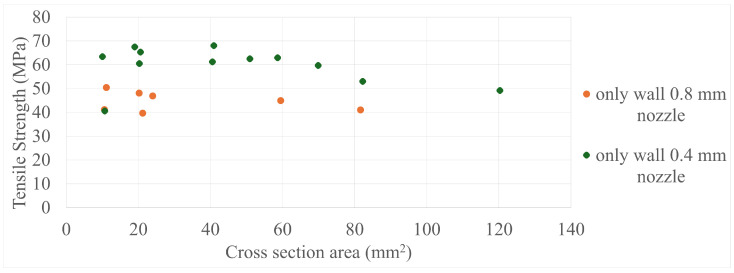
The tensile strengths of the only-wall 3D-printed specimens compared by their cross-section area.

**Figure 6:**
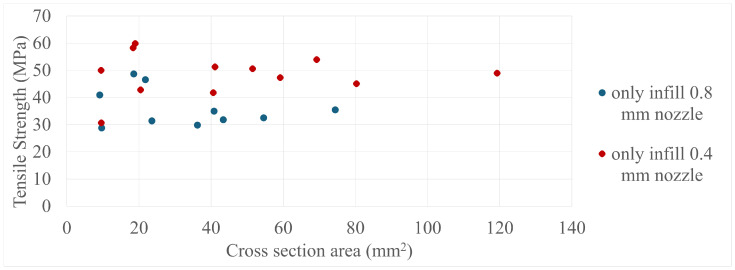
The tensile strengths of the only-infill 3D-printed specimens compared by their cross-section areas.

**Figure 7:**
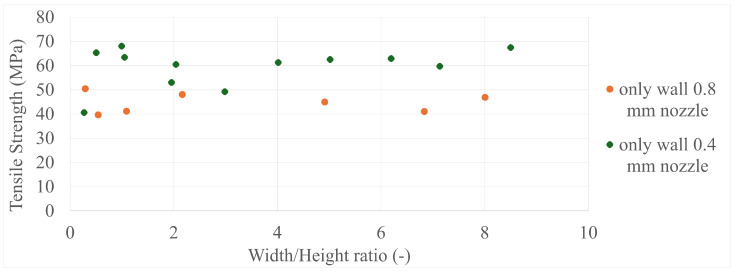
The tensile strengths of the only-wall 3D-printed specimens compared by their width/height ratios.

**Figure 8:**
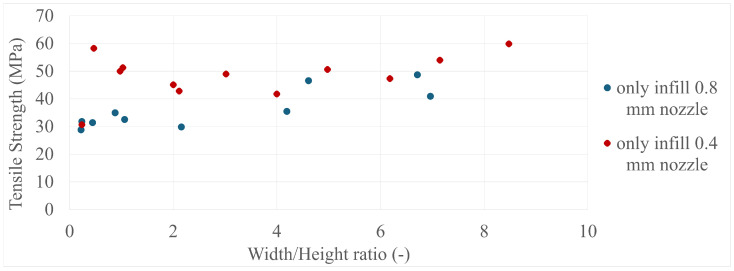
The tensile strengths of the only-infill 3D-printed specimens compared by their width/height ratios.

**Figure 9:**
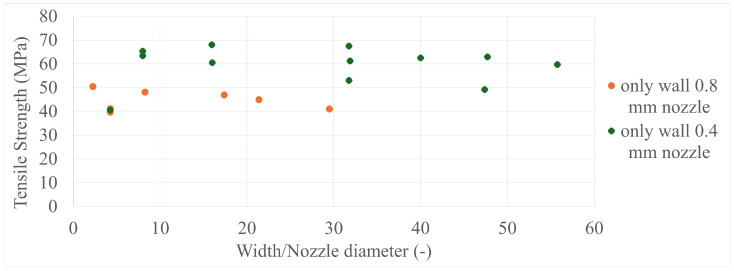
The tensile strengths of the only-wall 3D-printed specimens compared by their width/nozzle diameters.

**Figure 10:**
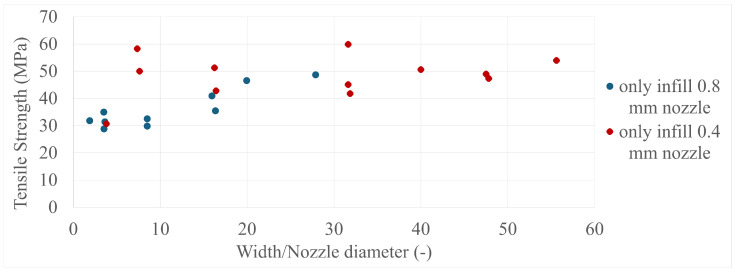
The tensile strengths of the only-infill 3D-printed specimens compared by their width/nozzle diameters.

**Figure 11:**
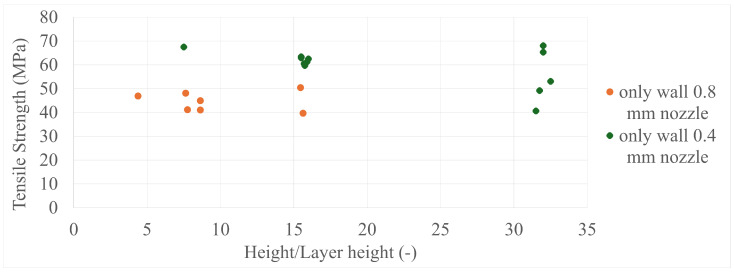
The tensile strengths of the only-wall 3D-printed specimens compared by their height/layer heights.

**Figure 12:**
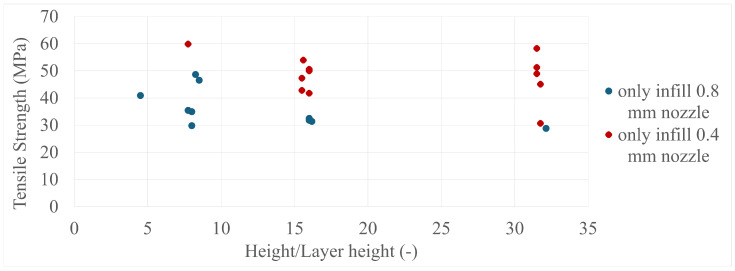
The tensile strengths of the only-infill 3D-printed specimens compared by their height/layer heights.

The final results in all four comparisons were very similar, with some variation between the specimens present; however, there was a lack of trend inside each specimen group as a whole. The corresponding values can be seen in Figure 5, Figure 6, Figure 7, Figure 8, Figure 9, Figure 10, Figure 11 and Figure 12. Each of the four specimen groups can be seen with the exact values and tensile strength deviation in Table 3, Table 4, Table 5 and Table 6.

The 0.4 mm nozzle specimens in Figure 5 show a drop in strength after 60 ${\mathrm{mm}}^{2}$; however, no other group showed such behaviour and it is well within deviation. Also, the 0.8 mm nozzle group in Figure 6 has three outliers in it, as they are 10–20 MPa stronger than the other ones with almost the same cross-section area; the fact they are within the strenght range of the 0.4 mm nozzle group further proves that they are outliers as the 0.4 mm nozzle specimens are always stronger. Calignano et al. [19] also investigated this material group from an other manufacturer with 0.4 mm nozzle 100% infill samples with a tensile strength of around 45 MPa and cross-section area around 10 ${\mathrm{mm}}^{2}$, which are in line with the lower end of these types of specimens presented above. Blok et al., 2018 [26] had examined the same kind of material group and structure, albeit with a larger 41.6 ${\mathrm{mm}}^{2}$ cross-section area specimen geometry and presented a much lower 33.5 MPa tensile strength value. The difference between the strength values presented in this paper and the aforementioned papers is most likely due to the numerous calibrations carried out beforehand listed in the prevoius section; the avarage tensile strength value for the 0.4 mm nozzle diameter and only-infill specimen group was 50 MPa. The comparison by cross-section area had been deemed the most important as it determines how many extruded lines a specimen is made up from and the relation between specimen size and nozzle diameter can be more easily understood.

In Figure 7, the 0.4 mm nozzle group seems to show a downward trend below 4 width/height ratio; however, above it, they settle around the 60 MPa mark, with a single outlier at around 0.25 width/height ratio. The 0.8 mm nozzle group again shows the 3 outliers at the last 3 values in Figure 8; they are again within the strength range of the 0.4 mm nozzle group.

The comparison by width/nozzle diameter in Figure 9 and Figure 10 shows more specimens with a single width/nozzle diameter, with the only-infill 0.8 mm nozzle still having the three outliers separate.

The height/layer height comparisons (Figure 11 and Figure 12), which describe the number of layers a specimen consists of, have more interesting content, as both the only-infill and only-wall 0.4 mm nozzle groups show the same behaviour as the three main groups gathered around the 8, 16, and 32 layer lines. They have a member that is among the highest strength values and the 32-layer line members have a greater spread than the 16-layer line members.

**Figure 13:**
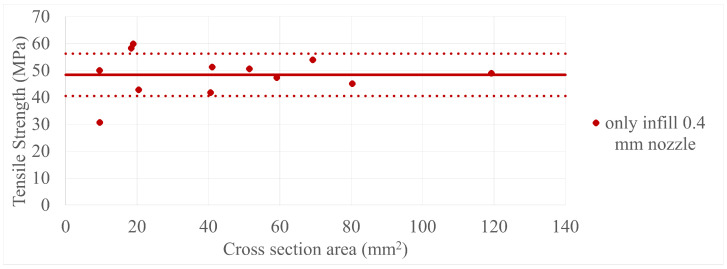
The only infill 0.4 mm nozzle specimen group with their mean value as a solid line and deviations as dotted lines.

**Figure 14:**
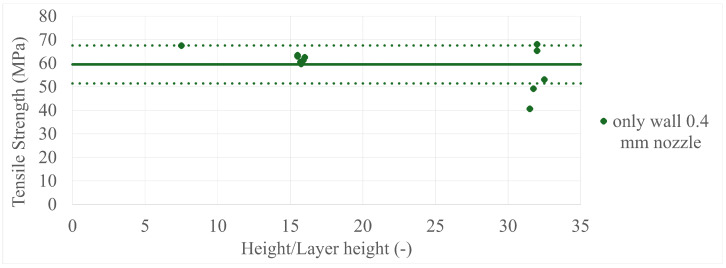
The only wall 0.4 mm nozzle specimen group with their mean value as a solid line and deviations as dotted lines.

**Figure 15:**
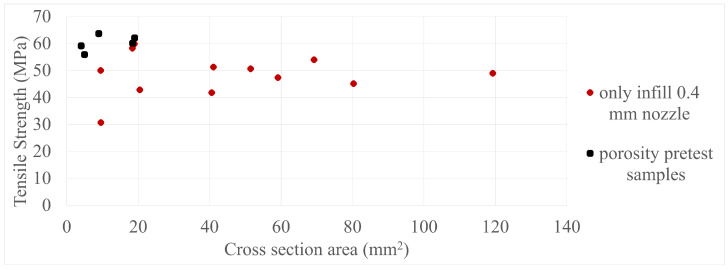
The tensile strengths of the porosity specimens included in their specimen group compared by their cross-section area.

**Figure 16:**
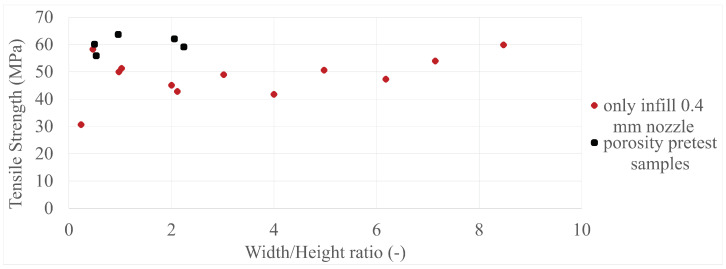
The tensile strengths of the porosity specimens included in their specimen group compared by their width/height ratios.

To further clarify that the above-presented values have no trend in them, two individual sample group can be observed in Figure 13 and Figure 14 with their mean values as a solid line and deviations as dotted lines. Every one of these is not included in the main paper as the paired up figures are already lengthy enough, so they are included in Appendix A. Also, the porosity pretest samples were added to their specimen group, which was the only-infill 0.4 mm nozzle group, to provide more data; the values can be seen in Figure 15 and Figure 16. The added data show that they are in line with the upper values of their group.

The exact values can be seen in Table 3, Table 4, Table 5 and Table 6 divided by the same groups as before. Almost all the tensile strength deviations are lower than 5% of their corresponding strength values with some exceptions like the 10.99 MPa deviation in Table 3 and the 5.11 MPa in Table 6.

## 4. Discussion

The initial aim for these experiments were to determine the effect of the geometry the various modified specimens have on the tensile strength based on the assumption that a body composed of numerous extruded lines of different sizes and shapes would be influenced by the shape and size of the lines that they are made of; however, the opposite case has been observed later during evaluation. All four specimen groups studied showed the same behaviour, where the geometry and size of the specimen had not influenced the tensile strength; rather, the different specimens’ strength values were around their mean value and within their deviation. The difference between the two nozzle sizes and structures used (wall and infill) is quite clear from the presented figures. All of the 0.4 mm nozzle specimens showed higher tensile strength values than their 0.8 mm nozzle counterparts, while all the specimens consisting of only walls consistently showed higher strength values than their counterparts consisting of only infills, typically in the range of 10–15 MPa. The only-wall samples’ higher strength can be easily explained by the fact that in them all the extruded lines (and by extension all the embedded carbon fibers) are parallel to the direction of tensile testing, while in the all-infill samples, it is only true for every second layer.

However, the clear difference in strength between the 0.4 mm and 0.8 mm nozzle diameter present in all four groups was a surprising result, as in one of our previous studies [22], the results showed the opposite behaviour in the 0° orientation samples, albeit in that study only a similar material group was used, glass-fiber-reinforced nylon, which can explain the discrepancy. Nonetheless, this discrepancy merits further examination of the impact the nozzle diameter has on tensile strength incorporating more material groups.

## 5. Conclusions

The conclusion can be drawn that the geometry and overall size of a specimen have no influence on its final tensile strength, making the comparison between studies using different specimen types valid. F Furthermore, an additional observation had been made that the specimens consisting of only walls and a 0.4 mm nozzle diameter showed greater tensile strength than their counterparts, only-infill and 0.8 mm nozzle diameter, respectively. Therefore, the strongest group measured was the only-wall 0.4 mm nozzle diameter group with a mean value of 60 MPa and a top value of 68 MPa.

## Figures and Tables

**Table 1:** The properties of the carbon fiber-nylon filament provided by the manufacturer.

Properties	Testing Method	Typical Value
Density	GB/T 1033	1.24 (g/${\mathrm{cm}}^{3}$)
Melt flow index	GB/T 3682	11.46 (275 °C/5 kg)
Tensile strength	GB/T 1040	140 (MPa)
Elongation at break	GB/T 1040	10.61 (%)
Flexular strength	GB/T 9341	140 (MPa)
Flexular modulus	GB/T 9341	4363 (MPa)
IZOD impact strength	GB/T 1843	18.67 (kJ/$m^{2}$)
Heat distortion temperature	GB/T 1634	155 (°C, 0.45 MPa)

**Table 2:** Tensile strength values for the porosity pretest groups.

Height/Width Ratio (−)	Cross-Section Area (${\mathrm{mm}}^{2}$)	Tensile Strength (MPa)	Tensile Strength Deviation (MPa)	Porosity (vol%)	Porosity Deviation (vol%)
0.50	18.42	60.11	3.63	4.80	1.61
0.53	4.94	55.92	0.90	6.16	1.43
0.96	8.94	63.70	5.97	5.37	1.48
2.06	19.00	62.10	6.13	3.24	0.63
2.24	4.02	59.14	7.81	3.71	1.15

**Table 3:** Tensile strength values for the only-wall specimens printed with a 0.4 mm nozzle.

Height/Width Ratio	Cross-Section Area	Tensile Strength	Tensile Strength Deviation
(−)	(mm^2^)	(MPa)	(MPa)
0.27	10.66	40.62	2.13
0.50	20.58	65.33	2.37
0.99	40.93	68.03	1.77
1.05	10.03	63.39	10.99
1.96	82.23	53.04	0.84
2.04	20.27	60.49	1.66
2.99	120.27	49.21	3.83
4.02	40.52	61.25	2.18
5.02	50.91	62.52	3.92
6.19	58.62	62.92	1.23
7.13	69.86	59.71	0.49
8.50	18.99	67.48	0.53

**Table 4:** Tensile strength values for the only-wall specimens printed with a 0.8 mm nozzle.

Height/Width Ratio	Cross-Section Area	Tensile Strength	Tensile Strength Deviation
(−)	(mm^2^)	(MPa)	(MPa)
0.29	11.10	50.47	2.67
0.54	21.18	39.69	0.74
1.09	10.54	41.21	2.36
2.16	20.17	48.11	0.55
4.91	59.46	44.99	3.67
6.84	81.60	41.06	0.53
8.00	23.95	46.92	1.18

**Table 5:** Tensile strength values for the only-infill specimens printed with a 0.4 mm nozzle.

Width/Height Ratio	Cross-Section Area	Tensile Strength	Tensile Strength Deviation
(−)	(mm^2^)	(MPa)	(MPa)
0.24	9.54	30.67	0.25
0.47	18.37	58.26	1.74
0.97	9.49	50.00	1.96
1.03	41.06	51.26	1.37
2.00	80.29	45.10	0.65
2.11	20.44	42.82	2.72
3.02	119.26	48.96	2.91
4.00	40.60	41.76	1.81
4.98	51.47	50.60	4.41
6.18	59.13	47.33	1.52
7.14	69.24	53.96	1.83
8.48	18.94	59.86	0.78

**Table 6:** Tensile strength values for the only-infill specimens printed with a 0.8 mm nozzle.

Width/Height Ratio	Cross-Section Area	Tensile Strength	Tensile Strength Deviation
(−)	(mm^2^)	(MPa)	(MPa)
0.22	36.20	28.84	1.24
0.24	9.63	31.84	2.22
0.44	18.53	31.44	0.86
0.88	9.11	34.99	1.62
1.06	43.35	32.55	1.71
2.16	21.75	29.85	3.32
4.19	40.80	35.49	5.11
4.61	54.54	46.59	0.26
6.71	74.39	48.72	0.43
6.96	23.56	40.95	0.84

## Data Availability

Dataset available on request from the authors.

## Supplementary Materials

The following supporting information can be downloaded at: https://www.mdpi.com/article/10.3390/polym17030401/s1.

## Appendix A

All the individual specimen groups visually enhanced with their mean value as a solid line and deviations as dotted lines.
